# Design of a consensus-based geriatric assessment tailored for older chronic kidney disease patients: results of a pragmatic approach

**DOI:** 10.1007/s41999-021-00498-0

**Published:** 2021-04-19

**Authors:** Carlijn G. N. Voorend, Hanneke Joosten, Noeleen C. Berkhout-Byrne, Adry Diepenbroek, Casper F. M. Franssen, Willem Jan W. Bos, Marjolijn Van Buren, Simon P. Mooijaart, the POLDER investigators, Arjan van Alphen, Noeleen Berkhout-Byrne, Fenna van Breda, Marjolijn van Buren, Henk Boom, Willem Jan Bos, Adry Diepenbroek, Marielle Emmelot-Vonk, Casper Franssen, Carlo A. J. M. Gaillard, Nel Groeneweg, Bettie Hoekstra, Nienke Hommes, Francoise Hoornaar, Hanneke Joosten, Joep Lagró, Elisabeth Litjens, Femke Molenaar, Simon P. Mooijaart, Aegida Neradova, Mike Peters, Wilma Veldman, Carlijn Voorend, Lidwien Westerbos, Carlijne Westerman - van der Wijden, Judith Wierdsma, M. Hemmelder, J. J. Homan van der Heide, K. Prantl, A. J. Rabelink, S. de Rooij, C. Stehouwer

**Affiliations:** 1 Department of Internal Medicine (Nephrology), Leiden University Medical Center, Leiden, The Netherlands; 2 Department of Internal Medicine, Division of General Internal Medicine, Section Geriatric Medicine, Maastricht University Medical Center+, Maastricht, The Netherlands; 3 Department of Nephrology, University Medical Centre Groningen, University of Groningen, Groningen, The Netherlands; 4 Department of Internal Medicine, St. Antonius hospital, Nieuwegein, The Netherlands; 5 Department of Nephrology, Haga Hospital, The Hague, The Netherlands; 6 Department of Gerontology and Geriatrics, Leiden University Medical Center, Leiden, The Netherlands

**Keywords:** Chronic kidney diseases, Clinical decision-making, Consensus development, Frailty, Aged, Geriatric assessment

## Abstract

**Purpose:**

Unidentified cognitive decline and other geriatric impairments are prevalent in older patients with advanced chronic kidney disease (CKD). Despite guideline recommendation of geriatric evaluation, routine geriatric assessment is not common in these patients. While high burden of vascular disease and existing pre-dialysis care pathways mandate a tailored geriatric assessment, no consensus exists on which instruments are most suitable in this population to identify geriatric impairments. Therefore, the aim of this study was to propose a geriatric assessment, based on multidisciplinary consensus, to routinely identify major geriatric impairments in older people with advanced CKD.

**Methods:**

A pragmatic approach was chosen, which included focus groups, literature review, inventory of current practices, an expert consensus meeting, and pilot testing. In preparation of the consensus meeting, we composed a project team and an expert panel (n = 33), drafted selection criteria for the selection of instruments, and assessed potential instruments for the geriatric assessment.

**Results:**

Selection criteria related to general geriatric domains, clinical relevance, feasibility, and duration of the assessment. The consensus-assessment contains instruments in functional, cognitive, psychological, somatic, patient preferences, nutritional status, and social domains. Administration of (seven) patient questionnaires and (ten) professional-administered instruments, by nurse (practitioners), takes estimated 20 and 40 min, respectively. Results are discussed in a multidisciplinary meeting including at least nephrology and geriatric expertise, informing nephrology treatment decisions, and follow-up interventions among which comprehensive geriatric assessment.

**Conclusion:**

This first multidisciplinary consensus on nephrology-tailored geriatric assessment intent to benefit clinical care and enhance research comparability for older patients with advanced CKD.

**Supplementary Information:**

The online version contains supplementary material available at 10.1007/s41999-021-00498-0.

## Key Summary points

**Aim:**

To propose a consensus-based geriatric assessment for optimizing both routine care and research in older patients with advanced chronic kidney disease.

**Findings:**

Using a pragmatic approach, we reached consensus on a suitable nephrology-tailored geriatric assessment to routinely identify major geriatric impairments in older patients with advanced chronic kidney disease. This geriatric assessment contains instruments in functional, cognitive, psychological, somatic, patient preferences, nutritional status, and social domains, and can be administered with patient questionnaires and professional-administered instruments by nurse (practitioners) in approximately 20 and 40 minutes, respectively.

**Message:**

We propose a consensus test set for standardized nephrology-tailored geriatric assessment, which is currently being implemented in multiple hospitals and studies, to benefit clinical care for older patients with advanced chronic kidney disease and enhance research comparability.

**Supplementary Information:**

The online version contains supplementary material available at 10.1007/s41999-021-00498-0.

## Introduction

Functional and cognitive impairment, frailty, and depression are highly prevalent in patients with advanced chronic kidney disease (CKD stage G4-G5; estimated glomerular filtration rate [eGFR] ≤ 30 mL/min/1.73 m^2^) [1–5], but are often unidentified [6]. These impairments are strongly associated with adverse health outcomes such as hospitalization and mortality [5, 7], and therefore relevant for risk stratification. Incorporating geriatric evaluation in routine nephrology care could better address older patients’ needs [8–11] and is recommended in recently published guidelines [12, 13].

Nephrology-specific geriatric assessment could be meaningful, first because the geriatric phenotype is often severely impacted by vascular problems, such as vascular cognitive impairment [14], which necessitates the use of instruments that are sensitive to these impairments. Second, due to the chronic nature of kidney diseases, nephrologists perceive to have a full geriatric picture of their patients’ status, but without objective measurements, impairments may still be overlooked [6]. Third and most importantly, broader knowledge of these impairments is important for (future) decisions regarding treatment for these vulnerable patients, and determining goals of ongoing care. Existing pre-decision care pathways mandate a geriatric assessment tailored to the older advanced CKD population. Fourth, a compromise between a full comprehensive geriatric assessment (CGA) and a brief screening (i.e., a modified geriatric assessment) might be a feasible solution to overcome practical barriers of implementation [8]. Comprehensive geriatric assessment (CGA), the cornerstone of geriatric medicine, has to conducted by a geriatrician and includes three elements; (i) thorough assessment of an older patient’s physical, functional, cognitive, and social capabilities, uncovering otherwise unnoticed impairments and detection of frailty, (ii) development of an integrated treatment plan, and (iii) evaluation of the progression of impairments and accordingly adjustment of the plan. CGA has shown to enable therapy adjustments and estimation of outcomes such as patient’s likelihood of living at home, limiting deterioration, and avoiding death [15, 16]. Yet, this systematic interdisciplinary process is time-consuming and therefore impracticable to use routinely in older CKD patients. Contrarily, geriatric screening with brief questionnaires is conduced to assess frailty and potential need for geriatric referral. However, this approach has lacked discriminating abilities to adequately recognize geriatric impairments or frailty in CKD patients [17]. Instead, we direct towards a geriatric assessment that aims to assess patients on all geriatric domains using validated questionnaires without necessary involvement of a geriatrician. Such a modified nephrology-specific geriatric assessment could be conducted by a trained nephrology nurse and be discussed in a multidisciplinary meeting, informing nephrology treatment decisions and follow-up interventions among which geriatric assessment identifies those patients who may benefit from CGA. In this model, the geriatrician adds valuable expertise on clinical judgement of frailty and appropriate interventions for older patients in addition to the nephrologists’ knowledge of the patient and the disease [18]. Clinicians have recognized that a standardized set of instruments could benefit these clinical purposes [5, 19].

In the absence of consensus on a uniform geriatric assessment for patients with advanced CKD, our aim was to propose a nephrology-tailored geriatric assessment, based on multidisciplinary consensus, useful in routine clinical care for older patients with advanced CKD.

## Methods

The current study aimed to reach agreement on a nephrology-tailored geriatric assessment (NGA) suitable to routinely identify major geriatric impairments in the target population, which was defined as older patients (≥ 65 years of age) with stage G4–G5 CKD. Ultimately, the test set should be ready to be implemented and evaluated in routine practice. Therefore, a pragmatic approach was chosen, which included focus group meetings to identify criteria for the assessment, literature review to identify potential instruments, questionnaires to inventory currently used instruments, an expert consensus meeting to ensure that the selection of tests was based on input from patients, clinical experience in nephrology and geriatrics, and pilot testing to ensure practicability. In preparation of the consensus meeting, we composed a project team and an expert panel, drafted selection criteria for the selection of instruments, and assessed potential instruments for the test set. After the consensus meeting, the set was pilot tested.

### Composition of the project team and expert panel

A multidisciplinary project team was formed, consisting of nephrologists (MB, WB, and CF), a geriatrician (SM), a nephrologist-geriatrician (HJ), nurse practitioners in nephrology (AD and NB), and a project leader (CV), to guide and prepare the consensus process. A multidisciplinary expert panel of 33 healthcare professionals was selected by an open purposive invitation for a meeting to find consensus on a preliminary test set. We invited medical doctors, nurses, and supportive disciplines experienced in the care for older kidney patients and/or with scientific experience in geriatric nephrology (Table 1).

**Table 1:** List of participants of the expert meeting 31^st^ of January, and input via round of comments

Discipline	Number of participants (*n* = 33)
Nephrologist (i.t)	12
Geriatrician (i.t)	5
Medical doctor (otherwise)	1
Nephrologist/geriatrician	2
Nurse practitioner (nephrology)	6
Nurse (nephrology)	3
Social worker	1
Physician assistant (i.t)	1
Other: medical information officer, project leader	2

*i.t.* in training

### Identifying potential selection criteria

A list of basic principles for geriatric assessment in nephrology was drafted by the project group, based on clinical and scientific expertise of the project team and general principles of geriatric assessment. The latter includes a holistic view of the patients’ unique needs and preferences, and assessment of health status in four domains (i.e., physical, cognitive, functional, and social), aiming to maximize self-reliance and quality of life.

In-depth input from older patients with advanced CKD, caregivers, and health care professionals experienced with conducting geriatric assessment in nephrology care was gathered in six focus group meetings. Purposively sampled CKD patients were included if aged ≥ 65 years, had an eGFR < 20 ml/min/1.73 m^2^), and had experience with geriatric assessment practices. Both patients with positive and negative experiences with NGA and with different (future) choices of treatment modality were invited, as were their caregivers. Detailed methods and overall results of these focus groups are published elsewhere [18]. Findings were included in the list of basic principles for geriatric assessment in nephrology which was presented and discussed in the expert meeting described below.

### Selection of potential instruments

Scientific use of geriatric tests was enumerated in published nephrology literature. We critically appraised previous systematic reviews [5, 7] and experience from (national ongoing) research cohorts in older patients with CKD G4-G5(D) [3, 6, 8, 11, 20, 21]. Tests with evidence-based associations with outcomes in older (CKD) patients were prioritized. In preparation of the consensus phase, two potential test sets were created to illustrate how the selection criteria and preconditions could work out in a practical test set.

Potential instruments for NGA were selected on the basis of current clinical and research practices. Clinical use was inventoried in 14 large Dutch academic and peripheral nephrology centers (comprising 25% of all Dutch nephrology centers). The centers were purposively selected based on their interest in implementation of geriatric assessment practices in nephrology. A questionnaire was sent to a nephrologist of each of the 14 centers by e-mail to ask for current and preferred geriatric screening instruments or assessment practices.

### Consensus meeting

The expert panel was invited to a meeting (January 31^st^ 2018) to discuss and reach consensus on a preliminary test set. Main aims of the meeting was to agree on (1) the proposed selection criteria, (2) the geriatric and clinical domains to be appraised, and (3) the selection of potential instruments. After the meeting, the preliminary test set was sent for a final round of comments to all attendees of the meeting, to experts who could not attend the meeting, and to a clinical neuropsychologist. Their feedback was discussed within the project group.

### Final test set

The final geriatric assessment was subsequently pilot tested by two experienced nurse practitioners from the project team (NB, AD), in two patients from different hospitals (patients aged 74 and 78 years, estimated glomerular filtration rate 18 and 26 ml/min/1.73 m^2^, respectively), to estimate administration time and patient and provider acceptability.

### Ethics information

This study is a narrative of a pragmatic consensus approach to improve routine clinical care, for which ethical approval is not applicable. Except for the qualitative part of the research using focus group discussions, which was approved by the Medical Research Ethics Committee United (MEC-U, Nieuwegein, The Netherlands, reference W17.127).

## Results

### Selection criteria

Table S1 presents the description of the generic purposes of the geriatric screening, criteria for selection of tests and questionnaires, criteria for feasibility, and duration of the assessment. Results of the focus group meetings [18] yielded five additional essential points: i.e., awareness of illiteracy, burden for patients, learning effect in case of repeated measurements over time, feasibility to conduct the NGA next to other local preferred instruments, and unpracticality of performing walking tests in the outpatient-clinic.

### Selection of instruments

The inventory of current geriatric screening instruments or assessment practices had a response rate of 100%. Supplementary Table S2 shows that 8 out of the 14 hospitals incorporated some form of geriatric screening or assessment in routine care for CKD G4–G5 patients, of which three for study purposes only. Among the hospitals, different screenings tests were used. Table S3 provides an overview of geriatric assessments as used in these hospitals and described in nephrology literature [3, 6, 8, 11, 20, 21].

### Reaching consensus on the domains and measures

In the expert panel meeting, first, the panel agreed on the selection criteria as proposed (i.e., basic principles for geriatric assessment in nephrology; Table S1), and underlined that feasibility was of utmost importance for use in routine care. Possible barriers were discussed: e.g., burden for patients, desirable answers, over testing, illiteracy and multi-ethnical patient population, duplication of locally used tests, unavailability of geriatricians, availability of time for conducting the assessment, and conflicting interests between pragmatic routine care and science. Second, the panel suggested three major changes after discussing two potential test sets. Primary, the panel agreed on including items of health-related quality of life (HRQoL) and patient preferences that correspond with the recently implemented Dutch patient-reported outcome measures (PROMs) for nephrology [22]. Secondary, the Montreal Cognitive Assessment (MoCA) was preferred over Mini-Mental State Examination (MMSE) for identifying mild cognitive impairment within the CKD population, especially because the latter is less in-depth and less discriminating [23, 24]. Tertiary, difficulties were recognized in standardized measurement of social domain. At the end of the meeting, consensus was reached on a preliminary test set including instruments for functional status, cognitive functioning, mood/psychological functioning, patient preferences, and frailty.

After the subsequent round of comments, the test set was changed on three aspects: instruments for assessing nutritional status and fall risk were added, and the Visual Association Test (VAT) was substituted by the Letter Digit Substitution Test (LDST) for the need of a more specific executive function test. The assessment comprises of patient questionnaires (seven instruments, including one caregiver questionnaire) and a test set administered by a professional (ten instruments).

### Items of the final consensus-based test set

Instruments are described below, and the main characteristics and cut-off points are summarized in Table 2. Supplementary Table S4 shows the predictive and diagnostic performance of tests in chronic kidney disease patients.

**Table 2:** Instruments and scoring of the consensus-based nephrology-tailored geriatric assessment

Domain	Instrument	Executed by	Explanation	Score/cut-off	Duration (minutes)
Functional status	Activities of daily living (Katz ADL-6) [25]	P	Grading of dependency on 6 functions, e.g., bathing, dressing, feeding	0–6 ^b^, ≥ 2 indicates dependency	2
	Instrumental Activities of daily living (Lawton) [26]	P	Grading of dependency on 8 more complex functions, e.g., ability to use telephone, housekeeping, medication	0–8 for women, 0–5 for men. Higher scores indicate more independency, no cut-off point	2
	Handgrip strength	I	Best of 3 repetitive measurements with dominant hand (i.e., no vascular access)	Reference value depending on age and gender [62]	4
	Fall risk assessment	I	1-year fall history and fear of falling	Yes/no; 1 (‘no fear’) to 10 (‘very afraid’)	1
Cognitive functioning	Montreal Cognitive Assessment [29]	I	Screening for mild cognitive impairment in 8 domains (i.e., visuospatial, naming, memory, attention, language, abstraction, delayed recall, orientation)	0–30, < 26 indication of cognitive impairment	10
	6-item Cognitive Impairment Test [31]	I	6-item screening for dementia, assessing orientation, attention, and memory	0–28, ≥ 11 indication of cognitive impairment	2–3
	Letter Digit Substitution Test [30]	I	Speed dependent task to measure speed of processing by matching letters to corresponding numbers provided in the key	Number of correct substitutions at 60 s; reference values depend on age, gender, education level [63]	5
Psychological status/mood	Whooley questions/Geriatric Depression Scale-15 [33, 34]	I^a^	Two initial question on depressed mood and anhedonia in the past month If yes on at least one question, 15-item GDS assesses presence and degree of depressive symptoms	Yes/no 0–15, (≥ 6 indicative of depression) [64]	1 5–7
	Life Orientation Test-Revised [35]	P	Dispositional optimism is measured by 10 items (including 4 filler items). Calculation of a total score, or the pessimism (reversed score on items 3, 7, 9) and optimism (items 1,4, 10) constructs separately	0 (‘strongly disagree’) to 4 (‘strongly agree’) 0–24 total score, or 0–12 per construct. Higher scores indicate more optimism, reference values depend on age and gender [65]	< 3
Patient reported outcome measures	HRQoL: 12-item Short Form Health Survey [41]	P	12 items on HRQoL providing a mental component summary (MCS) and physical component summary (PCS), using three- or five-point Likert scales	0–100, higher scores indicating better HRQoL	≤ 2 ^c^
	Dialysis Symptom Index [42]	P	Measuring symptom burden, by indicating presence of 30 or any other additional symptoms. If present, patients are asked to specify for the degree of bothersome	Yes/no, if yes 1 (‘not at all’) to 5 (‘very much’)	2–15 ^c^
Somatic status	Surprise question [40]	C	Clinicians response to the question: *“Would I be surprised if the patient died in the next 12 months?”* assessed by nephrologist, geriatrician and/or nurse (practitioner)	Yes/no	1
	Clinical Frailty Score [36]^,^	C	Clinical judgement on a visual and written chart with 9 graded pictures. ^d^	1 (‘very fit’) to 9 (‘terminally ill’)	< 1
	Charlson Comorbidity Index [37]	C	Comorbid conditions weighted for increased severity of the condition	1 to 6 points per condition, total range of 0–33	4
	Polypharmacy	C	Assessed by means of the total number of different medication for chronic use (i.e., for more than 2 weeks)	Use of five or more medications daily	2
Nutrition	Patient-Generated Subjective Global Assessment [45]	P P/I	Short Form includes 4 self-reported items on weight development, food intake, symptoms, and activities Complete PG-SGA: 5 additional items to be filled in by a clinician or dietician (diagnosis, age, metabolic stress, physical examination) to assess numerical score	Short Form only: 0–36 (≥ 6 indicates malnutrition) Complete PG-SGA: 0–52 (≥ 9 indicates malnutrition)[46]	1 5–10
Social	Caregiver burden: EDIZ-plus [47]	CG	Self-perceived burden from informal care measured in 15-statements to ‘agree’, ‘neither agree/nor disagree’ or ‘disagree'	1 point per question answered with ‘agree’; 0 (‘no burden’), 1–3 (‘minor burden’), 4–8 (‘moderate burden’), 9–15 (‘severe burden’)	5

*P* patient, *I* interviewer, *C* clinician, *CG* caregiver, *NA* not available, *HRQoL* health-related quality of life, *EDIZ* Ervaren Druk door Informele Zorg [Self-perceived burden from informal care]

^a^GDS can be either self-administered or by an interviewer, for more in-depth assessment interviewer-administered is preferred

^*b*^Score range 0–12 for the ternary-answering version

^c^12 min on average for both measures

^d^The initial seven-point scale version was expanded to a nine-point scale by the authors of the clinical fraily scale

#### Functional and performance status

Two instruments were selected to assess functional dependency. Katz Activities of Daily Living score (Katz-ADL-6) [25] was included to measure self-reported task for self-care on six functions of daily living activities; such as dressing and bathing. Whereas the Lawton scale for instrumental Activities of Daily Living (Lawton-iADL or IADL_8_) [26] measures more complex skills required for independent living in the community, such as handling finances and medication. Third measure of functional status is handgrip strength, which was considered important as it is associated with risk of commencing dialysis, and higher physical domain QOL scores [27]. Measure of gait speed was also considered as a sensitive and often used measure, but eventually not preferred above handgrip strength for practical reasons (i.e., gait speed assessment requires four to six meters of free space). Fourth, it was considered important to assess a person’s fall risk, as among older dialysis, patients falls are frequent and negatively impact HRQoL [28]. Two short questions were included on 1-year fall history and fear of falling. In summary, physical functioning is assessed by Katz-ADL-6, Lawton iADL, handgrip strength, and falls questionnaire in this final NGA.

#### Cognitive functioning

Three cognitive tests were included (i.e., MoCA, LDST, and six-item cognitive impairment test [6-CIT]), to ensure all unidentified cognitive deficits were captured. Besides, multiple tests allow future selection of the best fitting instrument to our population. The MoCA [29] is developed to measure cognitive decline in multiple cognitive domains, such as executive function, orientation, recall, and visuospatial ability. The LDST [30] is used in CKD G4-G5 patients [14] to measure the speed of processing general information. The 6-CIT [31] is a screening instrument for dementia, assessing only six items on orientation, attention, and memory. The 6-CIT is a feasible, acceptable, short, and simple instrument [32], which was a key reason for incorporating the test in the NGA set, and allowing further research to potentially replace MoCA and LDST by a shorter instrument.

#### Psychological functioning/mood

Depression is assessed in two steps. First, a validated two-item case-finding instrument is used, asking about depressed mood and anhedonia in the past month (‘Whooley-questions’) [33]. If at least one question is answered positive, the binary 15-item Geriatric Depression Scale (GDS-15) [34] will be assessed. GDS-15 is specifically designed for older persons by putting less weight into somatic symptoms as these may be part of comorbidity. The 10-item Life Orientation Test-Revised (LOT-R) [35] is used to assess dispositional optimism and pessimism. In summary, psychological functioning is assessed by the LOT-R and ‘Whooley-questions’ and GDS-15 in this final NGA.

#### Somatic status/clinical judgement

Consensus was reached about the inclusion of three different predictive measures of mortality: frailty, comorbidity, and the surprise question. Frailty is measured using the Clinical Frailty Scale (CFS) [36], i.e., clinical judgement score on a visual and written chart with nine graded pictures, varying from very fit tot terminally ill. Second, comorbidity is assessed with the Charlson Comorbidity Index [37], a score calculated by the patient’s comorbid conditions weighted for increased severity of the condition. It is the most common used validated prognostic index to predict mortality for CKD patients starting dialysis [38]. Finally, the surprise question (i.e., *‘Would I be surprised if the patient died in the next 12 months?’*) was included, since it performs as a predicter for death in CKD populations and because it is a simple and feasible instrument [39, 40].

#### Patient preferences and HRQoL

The 12-item Short Form (SF-12) and the Dialysis Symptom Index (DSI) were recently introduced as PROMs in Dutch Nephrological Care by the Dutch Kidney Patients’ Association, Dutch Federation of Nephrology, and Nefrovisie Foundation. SF-12 is a measure of eight domains of HRQoL, comprising of a mental- and a physical component summary score [41]. The DSI is a measure of symptom burden [42]. Patients are asked to indicate and rate the presence of 30 possible symptoms during the past week. Discussing symptoms could provide insights and guidance in symptom burden and management [43, 44].

### Other domains: nutrition, caregiver burden, and polypharmacy

Nutritional status is assessed by the Patient-Generated Subjective Global Assessment (PG-SGA) [45]. The PG-SGA contains a patient-questionnaire on intake, symptoms and physical activities (i.e., PG-SGA Short Form), and a professional part on subjective clinical judgement. The instrument can be used as a triage tool for nutritional interventions and to identify malnourished patients [46].

Caregiver burden is measured (optional) with a ‘self-perceived burden of informal care’ 15-item questionnaire (EDIZ-plus) [47], which can indicate overburdening of caregivers.

Polypharmacy, defined by use of five or more medications daily, is assessed by means of the total number of different medications for chronic use (i.e., for more than 2 weeks).

### Pilot testing

The geriatric assessment was pilot tested and took 20–30 min for the patient questionnaire and 30–45 min for the professional-administered set. Patient and professional acceptability was ensured as both nurse practitioners and patients did not have any remarks.

## Discussion

We propose a consensus-based nephrology-tailored geriatric assessment (NGA) suitable to routinely identify major geriatric impairments in older patients with advanced chronic kidney disease. The NGA contains instruments in functional, cognitive, psychological, somatic, patient preferences, nutritional status, and social domains. Selection of instruments resulted from focus group meetings with patients and professionals, literature evidence, inventory of current geriatric screening practices, consensus between clinicians from nephrology and geriatrics, and pilot testing. This first consensus-based geriatric assessment is intended for use in nephrology clinical practice, and can be seen as a first pragmatic step towards implementation of standardized geriatric practices in nephrology.

Other fields, such as oncology, have previously reported studies on the pivotable role of geriatric assessment for treatment decisions and plans [20, 48, 49], the development of new approaches for geriatric assessment [50–52], and consensus trajectories for these new approaches [53–55]. However, such initiatives are relatively new to the field of nephrology, where only a few NGA practices have been published. One example is the Renal Elderly Care Integration Project [8, 10] which presented a ‘modified geriatric assessment’ using similar domains compared to our proposed geriatric assessment. The main difference to our set is the choice of patient experiences (Renal Treatment Satisfaction Score and Distress thermometer) compared to our inclusion of PROMs measures [43], and the addition of instruments on nutritional status and caregiver burden. Compared to other geriatric assessment initiatives in nephrology, our set is more holistic than the presented set for the CGA-4-CKD and Renal Silver Program [20], and the Multi Prognostic Index [56], and more compact, benefitting use in routine practice, than sets designed for research [3, 6, 21]. Another recent initiative presented positive results on quality improvement by combined frailty screening and geriatric assessment practices [57].

For both the somatic and social domain, limited instruments were selected, under the assumption that regular anamnesis already addresses most somatic deficits. Our final consensus NGA may be considered a minimum data set, but is not all-inclusive. Additional domains and instruments may be of local interest and beneficial for the patient to assess; e.g., spiritual beliefs, life goals, physical fitness, cumulative illness rating scale, self-efficacy, health-literacy assessment, and patients’ outcome prioritizations. The proposed set consists of instruments that have been established in clinical or scientific use in aged populations, although some instruments (e.g., 6-CIT, LDST, LOT-R, CFS) should be further tested for use in CKD G4-G5 patients, as illustrated in Table S4. The geriatric assessment is aimed complementary to routine data collection as part of nephrology care (including, e.g., CKD classification, metabolic and cardiovascular parameters, cohabitation status, and history of smoking/alcohol use).

Implementation of geriatric assessment may differ locally, since health settings and structures diverge. Contrary to a time and labor-intensive CGA, the proposed NGA in this article is feasible within 1 h and could be assessed without involvement of a geriatrician. Rather, results are discussed in a multidisciplinary meeting, informing nephrology treatment decisions and follow-up interventions among which CGA and consultation of a geriatrician. The NGA could be conducted by a geriatric-trained nephrology nurse (practitioner) or by a partnership between geriatric medicine and nephrology (i.e., geriatrician co-management after screening for eligibility by geriatrician or triage nurse) as described elsewhere [18, 20]. However, appropriate geriatric training for clinicians to assess and manage geriatric conditions is advocated [3, 6, 8, 10, 20, 21]. Also, at least minimal geriatric involvement is recommended [55, 58]. Logistical difficulties of implementing geriatric assessment in new settings, like involving geriatricians and other team members being added to existing services, should not be under-estimated [50]. Factors, such as stretching budgets [50], shortage of geriatricians [55], and lack of knowledge on geriatric tools [8, 59], may hamper practical implementation.

Controversy still exists regarding the selection of patients for whom NGA is beneficial [50]. Due to considerable heterogeneity in the aging process, actual age may not always be useful. In oncology, patients’ age cut-off for assessment is often ≥ 70 years[53], but also those who are younger with age-related issues or concerns were recommended for assessment [55]. In nephrology, assessment has been reported from age of 65 [3, 6, 11, 21] or 70 years [8, 20], or younger if a patient is considered frail. Furthermore, timing of assessment is subject to further investigation. To optimally benefit the decision-making process, we advocate geriatric assessment in (late) CKD stage-G4 [20, 21], rather than at initiation or during dialysis [6, 8].

Strengths of our presented NGA test set is that it is a first consensus-proposal, based on current routine practices, with input from patients and a multidisciplinary expert panel. There are several limitations. First, this appraisal was neither based on a formal Delphi-method or nominal group technique, nor a systematic review of literature. Rather, our pragmatic consensus approach was seen as a first step to implementation of a standardized geriatric assessment in nephrology care. After evaluation of the NGA, subsequent Delphi-method may be useful in further implementation and development, as was done in oncology [54]. Second, bias is an inherent risk in consensus approaches, e.g., in selection of our expert panel. Although we aimed to represent all disciplines involved in CKD G4-G5 care, the panel was a selected group of Dutch professionals with special interest in the field of nephrogeriatrics, and may not reflect the general nephrology opinion. A third limitation is that the test set is less suitable in illiterate patients. Furthermore, although CGA has demonstrated benefits in other medical fields [15, 16], studies on its effectiveness are lacking. Though, use of a geriatric assessment was suggested to identify often undiagnosed problems [6], raise awareness for these problems among health care professionals [3, 18, 20], and enables treatment adjustment or tailored supportive interventions [48]. Besides, the information derived from standardized geriatric assessment can potentially be used to stratify patients into risk categories to better predict their outcomes on kidney replacement therapy or conservative care [5].

Future research is needed to evaluate the effectiveness and feasibility of implementation of the set. Also, value of NGA for decision-making, which is an extremely complex process, should be further explored. Therefore, insights in determinants of adverse outcomes and exploration of the prognostic capacity on outcomes are needed. Furthermore, (cost)effectiveness of geriatric assessment on key outcomes such as quality of life, hospitalization, treatment, and survival needs to be investigated in the older nephrology population. Along with the identification of standardized (preventive) clinical interventions for the management of geriatric impairments. As a first step, the NGA is currently implemented in 11 Dutch hospitals. In a pilot study [60], we will explore feasibility of implementation of the NGA in routine nephrology care and evaluate the included instruments (outcomes are expected in 2021). Experience and data from succeeding prospective cohort studies [61] could ultimately lead to prediction models to guide tailored treatment decisions or preventive interventions.

In conclusion, we propose a consensus-based nephrology-tailored geriatric test set to assess frailty, cognitive and functional status for older patients approaching kidney failure (CKD G4-G5), in accordance with (inter)national guidelines and suitable to routinely identify major geriatric impairments. Future research should investigate feasibility of implementation of this NGA and its value for decision-making trajectories for kidney replacement therapy, providing insights in determinants of adverse outcomes, and for improvement outcomes for older kidney failure patients.

## Supplementary Information

Below is the link to the electronic supplementary material.

Supplementary file 1 (DOCX 172 KB) Table S1: Basic principles of geriatric screening in nephrology. Table S2: Short questionnaire for inventory of current geriatric screening and assessment practices. Table S3: Inventory of use of instruments for geriatric screening and assessment in nephrology practice, study initiatives and published literature. Table S4: Diagnostic and predictive performance of consensus nephrology-tailored geriatric assessment test set for CKD G4-G5.

## Data Availability

Not applicable.
