# Supplementary material for: Design of a consensus-based geriatric assessment tailored for older chronic kidney disease patients: results of a pragmatic approach
Source: Eur Geriatr Med. 2021 Apr 19;12(5):931–42. doi: 10.1007/s41999-021-00498-0 (PMC8463384; doi:10.1007/s41999-021-00498-0)
Supplement: Supplementary file 1 — (DOCX 172 KB) Table S1: Basic principles of geriatric screening in nephrology. Table S2: Short questionnaire for inventory of current geriatric screening and assessment practices. Table S3: Inventory of use of instruments for geriatric screening and assessment in nephrology practice, study initiatives and published literature. Table S4: Diagnostic and predictive performance of consensus nephrology-tailored geriatric assessment test set for CKD G4-G5. [file 41999_2021_498_MOESM1_ESM.docx]

Supplementary Material

*Design of a consensus-based geriatric assessment tailored for older chronic kidney disease patients.* European Geriatric Medicine.
*Voorend CGN*, Joosten H, Berkhout-Bynre NC, Diepenbroek A, Franssen CFM, Bos WJW, Van Buren M, Mooijaart SP.**correspondance: [c.g.n.voorend@lumc.nl](mailto:c.g.n.voorend@lumc.nl)

Table S1: Basic principles of geriatric screening in nephrology

| Basic principles of geriatric screening | • Is aimed at creating improved awareness of geriatric focus areas for care providers and patients  • Provides input for the decision-making process and the conversation surrounding the treatment  • There is sufficient scope to complete this set in addition to the instruments already implemented locally  • Deviating results can form a reason to refer to the “geriatric specialist” for further investigation  • Significant changes over time can form a reason to re-evaluate treatment decisions with patients and their caregivers |
| --- | --- |
| Substantive selection of questionnaires and tools based on: | • Providing insight into all four of the classical geriatric domains (somatic disease and physical, cognitive/mental and social functioning)  • A combination of questionnaires and at least 1 objectively measurable physical parameter  • At least 1 measurement instrument/tool that provides insight into patient preferences and values (to be determined via focus groups)  • A sufficiently broad evaluation of the cognitive/mental domain (i.e. in several areas)  • Alignment with nationally implemented instruments, such as a safety management system  • Clinical experience from previous studies and/or care pathways involving the older persons  • Evidence from literature, preferably validated instruments in populations with CKD G4-G5, or from (older) populations from other specialisms (oncology/haematology, surgery, palliative care, etc.), including instruments required for the prediction of negative outcomes  • Clinical relevance (expert opinion, to be determined via focus groups and healthcare providers with experience of COPE study /GOLD study / best practice Groningen care pathway)  • Where possible, the experience from existing studies and care pathways in The Netherlands is used in the selection of tests: GOLD study, COPE study, Groningen care pathway and ICHOM  • Limitation of the learning effect where possible (considering the repetition factor over time) |
| Practical feasibility entails that the geriatric assessment: | 1. Can be completed in an outpatient setting 2. Does not form too much of a burden for the patient (to be determined via focus groups) 3. Aforementioned objective measurements are physically feasible for the CKD G4-G5 population 4. Can be completed uniformly in all participating Dutch centres 5. Can be completed by a trained nephrology nurse (after training), geriatric nurse or physician assistant in no more than [30-45-60] minutes (consult with focus groups and pilot centres) 6. In theory, a medically substantive consultation geriatric doctor should not be necessary for each screening (be aware: funding) |
| Considerations: | • What is the maximum time frame within which the geriatric screening should be able to take place?  • 30-45-60 minutes (in 2 sessions if necessary)  • In theory, perform geriatric assessment once a year:  • at start of dialysis  • monitor annually or biannually for a wait-and-see approach  • complete an extra screening set in the interim if indicated  • Geriatric medicine consultation is indicated in case of abnormal screening  • To be integrated based on the local capabilities and working arrangements |
| Input from focus groups: | • Pay attention to functional illiteracy in screening  • Take into consideration the taxability of older CKD G4-G5 patients  • Walking test impractical in outpatient setting  • Be wary of learning effect due to repetition factor  • Must be feasible in addition to the local instruments/preferences |

CKD, chronic kidney disease; ICHOM, International consortium for health outcomes measurement;
[Translated from Dutch by an certified (ISO 9001 and ISO17100) medical translation company.]

Table S2: Short questionnaire for inventory of current geriatric screening and assessment practices

| **No.** | **Question** | **Answers** | **Hospitals**  **(n=14)** |
| --- | --- | --- | --- |
| 1 | Is a geriatric assessment currently performed in the pre-dialysis care? | Yes, in routine care  Yes, study in routine care setting  No, geriatric assessment is done for study purposes only  No | n=5  n=3  n=3  n=3 |
|  | - 1. If yes, who performs this assessment (pre-dialysis nurse, geriatric nurse, physician, ...) | Geriatric department  Nephrology department  Combined | n=2  n=5  n=1 |
|  | - 1. If yes, which tests are used for this assessment? | *See Table S3* | |
|  | - 1. If yes, how is this assessment integrated in the standard pre-dialysis care? | Integrated/combined with regular visit to outpatient clinic  Separate patient visit | n=5  n=3 |
| 2 | What cooperation currently takes place between geriatric care and nephrology? | 1. Internist-geriatrician and nurse (practitioner) are part of a multi-disciplinary kidney failure team, or the multi-disciplinary consultation of the pre-dialysis outpatient clinic  2. By nurses/nursing specialists in pre-dialysis and geriatric care  3. Geriatric outpatient clinic separate from kidney failure carousel, but the plan is to combine these  4. Short communication lines with clinical geriatric care and separate slots at clinical geriatrics outpatient clinic  5. No structural consultation, referral from nephrology department  6. None at the moment, agreement to have older pre-dialysis patients seen by geriatric specialists in the near future  7. Good cooperation, set-up of pre-dialysis outpatient clinic supported by both geriatric specialists and nephrologists | n=2  n=2  n=1  n=1  n=4  n=5  n=1 |
|  | Are there nursing specialists and specialist dialysis nurses employed by the department who play a role in the pre-dialysis care? | Nephrology nurse practitioner  Pre dialysis nurse  Geriatric nurse  Geriatric nurse practitioner | n=4  n=7  n=1  n=2 |
| 3 | Do you expect any logistical problems in the implementation of a standard geriatric assessment in pre-dialysis care? (based on the duration of the assessment of maximum 1 hour, for patients aged 65+ years) | | |
|  | 1. If yes, which problems? | 1. Staffing, particularly in clinical geriatrics  2. Logistics: Outpatient planning and spaces  3. Time: 1 hour for a first intake is long  4. Multi-disciplinary consultation too full for discussion of assessment, need to create inventory of the number of patients  5. Extra appointment for patient, carousel outpatient appointments not possible  6. Nurse and nephrologist are not trained  7. Database entry | |
|  | 1. If no, what is the plan of action? | 1. Expansion of standard geriatric assessment from patients 70+ to 65+  2. Information supplied by nephrology, screening by nursing specialist under supervision (resident) geriatric specialist, discussion in multi-disciplinary meeting  3. Same as COPE study: combined appointment with pre-dialysis and geriatrics, testing by geriatric nurse or pre-dialysis nurse  4. Consultation with internist-geriatrician in kidney failure carousel, nursing specialist provides information, specialised nurse performs GA  5. Probably as part of standard patient care  6. Possibly delegate care from clinical geriatrics to dialysis nurse and social worker, evaluation with clinical geriatrics in multi-disciplinary meeting  7. Separate appointment for extra geriatric assessment and open outpatient clinic for this purpose  8. Plan will be worked out with geriatric specialists | |
| 4 | Do you foresee any problems with online data entry in the RENINE database?  (assuming 1/2 hour time budget per patient, aim to see at least 20 patients per hospital over a period of one year) | No  Possible  Yes  Missing | n=10  n=1  n=2  n=1 |

Table S3: Proposed geriatric assessment test set compared to other sets reported in literature as used in nephrology clinical practice. (continued on next page)

|  | **Proposed nephrology-tailored geriatric assessment (POLDER)** | **Renal Elderly Care Integration Project[1]** | **Parlevliet et al. [2]** | **CGA-4-CKD Program[3]** | **Renal Silver Program[3]** |
| --- | --- | --- | --- | --- | --- |
| **Characteristics:** |  |  |  |  |  |
| Duration | One hour or less | One hour | One hour (+ one hour q.) | <10 minutes | 45 minutes |
| Assessed by | NP, dialysis/geriatric nurse, team | Specialized nurse (team) | Research nurse | Geriatrician or nurse | Nephrologist or NP |
| Target population | CKD G4-G5  >70 years of age | Dialysis patients (HD/PD)  >70 years, 60-70 years at indication, (n=118) | Dialysis patients (HD/PD)  >65 years, (n=50) | CKD, not in geriatrics clinic,  >70 years, (n=33) | Advanced CKD  >75 years, (n=35) |
| Setting | Routine practice | Routine practice: outpatient clinic / dialysis unit | Home visit | In CKD clinic | In CKD clinic |
| **Domain:** |  |  |  |  |  |
| Functional status | *Katz Activities of Daily Living (6 item)*  *Lawton i-ADL* | *Personal care: help required for Activities of Daily Living* | *Katz Activities of Daily Living  Modified Katz index for iADL*  *Neurosensory impairments* | *Activities of Daily Living ^a^*  *i-ADL ^a^* | *Activities of Daily Living ^a^* |
|  | *Handgrip strength*  *Fall risk: 1-year fall history, fear of falling.* | *Mobility: aids*  *Fall history* | *Walking aid*  *≥Two falls in past three months* | *Mobility (walking ability)*  *Falls (prior 12 months)* | *Mobility (walking aid)*  *Falls (prior 12 months)* |
| Cognitive functioning | *Montreal Cognitive Assessment*  *Six-item Cognitive Impairment Test*  *Letter Digit Substitution Test* | *Abbreviated Mental Test Score*  *Clock drawing* | *Mini Mental State Examination*  *IQCODE-SF*  *NPI-q*  *Confusion Assessment Method* | *Dementia diagnosis or   Mini-Cog test* | *Dementia diagnosis or   Mini-Cog test* |
| Mood | *Geriatric Depression Scale*  *Optimism check* | *Distress thermometer*  *Social: (anamnesis)*  *Environmental: (anamnesis)* | *Geriatric depression scale-15*  *Loneliness: De Jong-Gierveldschaal* |  |  |
| Quality of life and patient preferences | *Short Form-12*  *Dialysis Symptom Index* | *Renal Treatment Satisfaction*  *Score (Patient experience)* | *EuroQol-6D, including VAS* |  |  |
| Somatic functioning (Clinical judgement) | *Surprise question*  *Clinical Frailty Score*  *Charlson Comorbidity Index*  *Polypharmacy* | *Clinical Frailty Score* | *Charlson comorbidity index*  *Polypharmacy: number of   different medications.* | *SOF (Frailty)* |  |
| Nutrition | *PG-Subjective Global Assessment* | *-* | *Short Nutritional Assessment Questionnaire*  *Body Mass Index* |  |  |
| Caregiver burden | *EDIZ-plus* | *-* | *EDIZ* |  |  |
| Other: |  |  | *Pain (VAS)*  *Decubitus, Constipation, Incontinence* | *Urinary incontinence* |  |

Purple coloured: indicates a different test than included in the proposed nephrology-tailored geriatric assessment

Abbreviations: CKD, chronic kidney disease; EDIZ, Ervaren Druk door Informele Zorg’ [Self perceived burden from informal care]; HD, hemodialysis; iADL instrumental Activities for Daily Living; NPI-q, Neuropsychiatric Inventory-questionnaire; NP, nurse practitioner; PD, peritioneal dialysis; PG, patient generated; SOF, Study of Osteoporotic Fracture frailty criteria; VAS: visual analogue scale
^a^ unspecified which instrument was used.

Table S3 (continued)

|  | Soysal et. al.[4] | COPE [5] | GOLD[6] | Clinical nephrology practice in the Netherlands |
| --- | --- | --- | --- | --- |
| **Characteristics:** |  |  |  |  |
| Duration | Not specified | 3 hours | 60-90 minutes | *(see supplementary Table S1)* |
| Assessed by | Not specified | Nurse practitioner or geriatric nurse | Research nurse |  |
| Target population | Dialysis patients (HD)  >65 years, (n=121) | CKD patients with eGFR<20  >65 years, (n=157) | Initiating dialysis or CC  >65 years, (n=285) |  |
| Setting | In clinic, after dialysis session | In CKD clinic | Home visit |  |
| **Domain:** |  |  |  |  |
| Functional status | *Basic ADL*  *IADL* | *Groningen Activity Restriction Scale*  *Lawton iADL* | *Katz-ADL*  *Lawton iADL* | *Katz-ADL*  *Lawton iADL* |
|  |  | *Handgrip strength*  *Gait speed*  *Short Physical Performance Battery* | *Handgrip strength*  *Falls*  *Four meter walking test*  *Timed up and go*  *Four Test Balance Scale* | *Timed up and go*  *Falls*  *Gait speed*  *Handgrip strength*  *Short physical performance battery* |
| Cognitive functioning | *Mini Mental State Examination* | *Mini Mental State Examination*  *Clock drawing*  *15- WVLT*  *Stroop Colour Word Test*  *Trail making test (A&B)*  *Visual Association Test*  *Letter Digit Substitution Test*  *Assessment of numeracy* | *Mini Mental State Examination*  *Clock drawing*  *Enhanced Cued Recall*  *Semantic Fluency Test*  *Interview of Detoriation in Daily life ^c^*  *IQCODE ^c^*  *Neuro-Psychological Inventory (mood) ^c^* | *Mini Mental State Examination*  *Montreal Cognitive Assessment (MoCA) ^b^*  *Cambridge Cognitive Examination*  *Six-item Cognitive Impairment Test* |
| Social functioning / Mood | *Geriatric Depression Scale-15* | *Geriatric Depression Scale-15* | *Geriatric Depression Scale-15* | *Geriatric Depression Scale-2/15* |
| Quality of life and patient preferences |  | *RAND-36* | *EuroQol-5D* | *Outcome Prioritization Tool (treatment goals)*  *EuroQol-5D*  *Visual analogue scale* |
| Somatic functioning (Clinical judgement) |  | *Fried frailty indicator*  *Charlson comorbidity index* | *Surprise question*  *Groningen Frailty index*  *Fried Frailty index*  *CIRS-G*  *Overall condition (VAS)* | *Rockwood Clinical Frailty Score*  *Groningen Frailty Indicator*  *Geriatric-8*  *Charlson Comorbidity Index*  *Surprise question* |
| Nutrition | *Mini Nutritional Assessment*  *Body Mass Index* | *Short Nutritional Assessment Questionnaire* | *Mini Nutritional Assessment* | *Mini-Nutritional Assessment*  *Body Mass Index*  *(Anamnesis by dietician)* |
| Caregiver burden |  | *EDIZ-plus* | *EDIZ* | *(Hetero-anamnesis)* |
| Other: |  | *Cantril’s ladder, Pain score, Anxiety score*  *Illness perceptions questionnaire* |  |  |

Purple coloured: indicates a different test than included in the proposed nephrology-tailored geriatric assessment

Abbreviations: ADL, Activities of Daily Living; iADL, instrumental Activities of Daily Living; CIRS-G, Cumulative Illness Rating Scale- Geriatric version; CKD, chronic kidney disease; EDIZ, Ervaren Druk door Informele Zorg [Self-perceived burden from informal care]; eGFR, estimated glomerular filtration rate; HD, hemodialysis; VAS, visual analogue scale; IQCODE, Informant Questionnaire on COgnitive DEcline.
^b^ Clock drawing is included in the Montreal Cognitive Assessment, *^c^* subjective cognition tests filled in by caregiver,

Table S4 : Diagnostic and predictive performance of consensus nephrology-tailored geriatric assessment test set for CKD G4-G5

| **Domain** | Subdomain | Instrument | Validated in CKD patients | Purpose and construct to assess | Performance | Validation cohort / source |
| --- | --- | --- | --- | --- | --- | --- |
| **Functional** | Functional dependency | Katz ADL-6[7] | Partly: in  HD patients | Assesses activities of daily living Predicting mortality | - Despite frequent use of Katz’ index, evidence on validity and reliability is limited [8, 9], terminology inconsistently used, and different versions (5/6/7 items) exist[8].  - Reliability rated questionably positive, validity and responsiveness both moderate or unknown for Katz 6-item version.[8]  - Construct validity is established with SF-36 physical and mental function domains at 1-month. Criterion predictive validity on short-term mortality but not longer-term mortality 3-6 months.[10]  - Good internal consistency (Cronbach’s alpha’s: 0.93 and 0.94) for 10-item and 5-item version[11], good acceptability and reasonable criterion validity for the former version.[9]  - Limited utility because of ceiling effects[12] | Generally widely used [8, 13, 14]  Older patients (55-74 years)[9]  Systematic review in older (>60 year) community-dwelling population [8]  Systematic review in critically ill [10]  General population sample[9]  Hemodialysis patients[11, 12] |
|  |  | i-ADL_8_ Lawton [15] | Partly: in  HD patients | Assesses instrumental activities of daily living  Predicting mortality | - Good internal consistency (Cronbach’s alpha 0.87).[11] Limited utility due to ceiling effects[12] - Predicting post-hospital and cumulative mortality, conflicting results for in-hospital mortality[10].  - Dependency (combined Katz ADL-5 and Lawton iADL_8_ scales) were predictor of mortality in HD and associated with dialysis withdrawal and HRQoL[16] | Generally widely used [13]  Hemodialysis patients [11, 12, 16] |
|  | Hand grip strength | Hand grip strength | Yes | Assesses muscle strength  Predicting mortality and outcomes | - Good to excellent test-retest reliability (ICC >0.80). High variability of minimal detectable change between studies (14.5-98.5%)[17]  - Predictive for functional status (pooled ratio 1.78 [95% CI 1.28–2.48] for high vs low handgrip strength, and 0.95 [95% CI 0.92–0.99] for handgrip strength as a continuous variable) and mortality (pooled HR 1.79 [95% CI 1.26–2.55] for high vs low handgrip strength and 0.96 [95% CI 0.93–0.98] for continuous handgrip strength).[18]  - Predictive for lower all-cause mortality (low versus high hand grip strength [RR 1.88, 95%CI 1.55-2.33], and per 1kg increase [RR 0.95, 95%CI 0.93-0.97]) in dialysis patients.[19]  - Associated with HRQoL scores (+0.13 [95%CI 0.01-0.24] in total score per 1kg) and decreased risk for commencing dialysis in CKD patients (aHR 0.89 [95%CI 0.84-0.96]) [20] or reaching composite end points in male CKD G3b-G5 patients (aHR 3.72 [95%CI 1.03-13.41)[21]  - Sensitivity of outcome predictability was 95% and 88%, and specificity 50% and 58%, for cut-off scores of 20.15kg and 10.15kg in resp. men and women [21] | Systematic review including older patients[17]  Systematic review: older patients (60+ years)[18]  Systematic review: HD/PD patients[19]  CKD G1-G5 not on dialysis[20, 21]  CKD G3b-G5[21] |
|  | Fall risk | Fall risk assessment | No | Fall history and fear of falling | No appropriate fall risk screening tool is available[22] | Systematic review[22] |
| **Cognitive** | Cognitive decline | MoCA[23] | Yes | Assesses (mild) cognitive impairment | - Good internal consistency (Cronbach alpha 0.83). High test-retest reliability (correlation coefficient =0.92 over mean 35 ±17.6 days). Content validity established with Mini-Mental State Examination (r = 0.87, p<0.01) [23], and concurrent validity established with a neuropsychological test battery in HD patients.[24]  - No consensus on the best cutoff point [25]. Original instrument uses cut-off of ≤25 for detecting mild cognitive impairment (sensitivity 90%, specificity 87%, PPV 89%, NPV 91%)[23] In HD patients: good sensitivity (77% and 87%) and specificity (79% and 57%) for cutoff scores of ≤24 and ≤25 respectively. AUC was optimal for cutoff scores of ≤24 (76%, 95%CI 0.60-0.87), with PPV 0.88, NPV 0.61.[24] Similar to findings in meta-analysis for older patients (best cutoff ≤24 yielded sensitivity 80%, specificity 81%, AUC 0.85 95%CI 0.82-0.87; while cutoff ≤25 yielded sensitivity 90%, specificity 57%)[26] | Patients with mild cognitive impairment and healthy controls[23]  Hemodialysis patients[24]  Systematic review: CKD including pre-dialysis populations [25]  Systematic review: older patients (60+ years)[26] |
|  |  | LDST[27] | No | Assesses psychomotor speed | - High test-retest correlation (r=0.88) and manageable by older persons[28]. | Older persons (70-82 years)[28] CKD late G4-G5 patients [29] |
|  |  | 6-CIT[30] | No | Assesses cognitive impairment | - Concurrent validity established with MMSE[31, 32] and expert diagnosis of delirium/dementia[33]  - Adequate to good sensitivity (73%-83%) and good specificity (>90%), excellent AUC (>0.91) for cognitive impairment (cutoff score ≥11) according to the MMSE.[31, 34] PPV (0.83), NPV (0.98)[31]  - Promising instrument, but further robust validation studies are required, including the best cuttoff[32]  - Associated with adverse outcomes, and a predictor of length of stay (adjusted OR 1.54, 95%CI 1.1-2.1), new institutionalization (adjusted OR 3.45, 95%CI 1.9-6.3) and in-hospital mortality (adjusted OR 3.11, 95%CI 1.2-8.0) for older acutely hospitalized patients[35] | >70 year old general hospital patient population[31]  Review of primary and secondary care settings[32]  Referrals to outpatient dementia service[34]  Emergency department attendees (>70 years)[33]  Older (>70 year) acutely hospitalized or emergency department patients[35] |
|  | Depression | ‘Whooley-questions’[36] | No | Screening for /diagnosing depression | - Adequate to high sensitivity (79-96%), but low specificity (54-57%), LR+ 1.9-2.2, LR- 0.07-0.37, AUC 72-82% [36] [37] | Adult primary care patients [36]  Older primary care and hospitalized patients (60+ years)[37] |
| **Mood** |  | GDS-15[38] | No |  | - Adequate sensitivity (79%, 95%CI 0.70-0.86), specificity (77%, 0.73-0.81) in meta-analysis. Test accuracy LR+ 3.41, LR- 0.29. Diagnostic odds ratio of 12.4, AUC 84%, at a cutoff score of 5/6.[39]  - Pooled sensitivity (0.89 95%CI 0.80-0.94) and specificity (0.77, 0.65-0.86) for a cutoff score of 5. [40] | Meta-analysis of studies among older in-patients (60+ years) from general hospitals[39]  Older population (55+ years) in primary care, secondary care, and community settings[40] |
|  | Optimism | LOT-R[41] | No | Assessing optimism and pessimism | - Acceptable reliability for subscales of optimism and pessimism (Cronbach alpha 0.70, and 0.74 respectively), questionable for the total score (alpha 0.68). Convergent validity is assessed with other psychological scales for depression, anxiety, pain, disability, self-reported state of health and multiple domains of life satisfaction (Pearson correlations presented) [42]  - Optimism was associated with pro-health behaviours in kidney transplantation patients[43] | General population [42]      Kidney transplantation patients[43] |
|  |  |  |  |  |  |  |
| **PROMs** | HRQoL | SF-12[44]  SF-12 (continued) | Yes | Assessing HRQoL (physical and mental component scale)  Predicting mortality and hospitalization | - Validated to detect change in HRQoL physical component score (PCS) and mental component score (MCS) (ICC with SF36 0.83 and 0.90 respectively)[45].  - Good evidence for reliability for PCS and MCS (Cronbach alpha=0.89 and 0.90, respectively) and test-retest reliability (ICC 0.76 and 0.74 respectively). No floor and ceiling effects. Acceptable convergent validity assessed.  Higher HRQoL PCS and MCS were associated with decreased mortality (resp. aHR -2.4% and -1.2% per incremental point) and first hospitalization (resp. aHR -1.2% and -0.6% per point).[45, 46] | HD/PD patients [45]  Hemodialysis patients [46, 47] |
|  | Symptoms | DSI[48] | Yes | Assessing symptom burden | - Content validity established, and reliable (Cronbach alpha 0.90). [49] Good test-retest reliability (total agreement mean 0.80±0.09). Kappa statistics for the items ranged from 0.06 to 0.90 (mean 0.48±0.22).[48]  - Responsiveness, smallest detectable change and minimal important change need further investigation[49]. | Advanced CKD[49] and hemodialysis patients[48] |
| **Somatic** | Clinical judgement | Surprise question (binary)[50] | Yes | Predicting mortality | - Poorly to modestly predictive performance for death at 6 to 18 months.  - Pooled prognostic characteristics: sensitivity 67% (95%CI 0.56–0.77), specificity 80% (0.73–0.86), positive likelihood ratio 3.4 (2.8–4.1), negative likelihood ratio 0.41 (0.32–0.54), positive predictive value 37% (0.30–0.45) and negative predictive value 93% (0.91–0.95). Adequate discrimination in patients with noncancer illness (AUC = 0.77, 0.73–0.81)[51] | Meta-analysis (including 7 dialysis populations out of 11 noncancer illness studies, additional to 5 cancer studies) [51] |
|  |  |  |  |  | - Sensitivity 66% (95% CI, 0.49-0.80), specificity 68% (0.63-0.73). Predictor of mortality (aHR 3.3; 95% CI, 1.8-6.0). Moderate inter-rater reliability (Krippendorff’s alpha = 0.58, 0.42-0.72), good test-retest correlation (Pearson coefficient = 0.66, 95% CI, 0.58-0.75)[52] | Outpatient CKD G4-G5 not on dialysis, aged ≥ 60 years. [52] |
|  | Frailty | CFS[53] | No | Predicting mortality, entry into institutional facility~~,~~ and length of stay | - Confirmed construct validity with multiple tools for measuring degree of frailty (Pearson coefficient 0.80, p < 0.01). High reliability between 2 ratings (ICC 0.97, p < 0.001). Interrater reliability not assessed. Adequate prediction of entry into institution (AUC = 0.75), and 18- month (AUC = 0.77) and 5-year mortality (AUC = 0.70). Calibration not assessed. | Community-dwelling older people[53] |
|  |  |  |  |  | - Adequate prediction of in-patient mortality (AUC = 0.72, 95%CI 0.69 – 0.75, p<0.001). Moderate prediction of transfer to geriatric ward (AUC = 0.68, 0.66 to 0.71, P<0.001) and length of stay ≥10 days (AUC = 0.62, 0.61 to 0.64, P<0.001). [54] | Emergency admissions of people aged ≥75 years[54] |
|  |  |  |  |  | - Associations between CFS and mortality in pre-dialysis (aHR 1.35, 95%CI 1.16 – 1.57) [55] and incident dialysis patients (aHR 1.32; 95%CI, 1.15 to 1.52)[56] indicates potential prognostic significance. | Patients referred to pre-dialysis eduction[55]  Adult patients on incident dialysis in tertiary centre[56] |
|  | Comorbidity | CCI[57] | Yes | Predicting mortality | - Acceptable discrimination performance (c-statistic = 0.74, 95%CI 0.65-0.83). Calibration was lacking reporting. Among prognostic indices, CCI delivered best and most discrimination performance, and is most commonly used. [58] | Patients starting dialysis (meta-analysis) [58] |
|  |  |  |  |  | - Poor discrimination (c-statistic = 0.63, 0.63-0.64) for one year mortality, calibration not reported. [59]  - Association between CCI and mortality in pre-dialysis patients (aHR 1.18, 1.05-1.34). [55] | Patients eGFR≤45ml/min/1.73m^2^ (large retrospective cohort) [59]  Patients referred to pre-dialysis eduction[55] |
|  | Polypharmacy | Five or more medications daily | NA | NA | Polyfarmacy definitions are variable, ranging from ≥2 to ≥ 11 medications daily. Most commonly definition is: the use of ≥5 medications daily.[60] | Systematic review of definitions[60] |
| **Nutrition** |  | PG-SGA[61] | Yes | Assessing malnourishment | PG-SGA score (cut off score ≥4, defined by PG-SGA global rating B=moderately malnourished or C=severely malnourished): AUC 93% (95%CI 0.90-0.97), sensitivity 90%, specificity 83%, accuracy 85% [62].  Complete PG-SGA (cut-off score ≥9, as defined by SGA≥9): sensitivity 83%, specificity 92%, PPV 71%, NPV 96%., poor reliability (Cronbach alpha=0.52), satisfactory internal reliability (standardized item alpha coefficient=0.73) [63].  PG-SGA Short Form as stand-alone questionnaire (using cut-off score ≥6, as defined by a complete PG-SGA≥9): sensitivity 78% (95%CI 0.64-0.88),specificity 94% (0.86-0.98), PPV 91% (0.79-0.98), NPV 84% (0.74-0.92), overall accuracy (AUC: 87%).[64] | CKD patients (inpatients, outpatients combined, including dialysis patients) [64] and HD patients[62, 63]. |
| **Social** | Caregiver burden | EDIZ-plus[65] | No | Assessing self-perceived pressure of informal care | Validated: average scalability (H= 0,46), good reliability  (Rho=0,87) and a good convergent validity (r=0,70)[65] | Informal carers for older persons (65+ years of age), physically disabled, institutionalized patients, mentally disabled, psychiatric problems [66] |

Abbreviations: NA, not applicable; (i)-ADL, (instrumental) activities of daily living; AUC, area under the curve; aHR, adjusted hazard ratio for mortality; CCI, Charlson comorbility index; CFS, clinical frailty scale; CKD, chronic kidney disease; DSI, dialysis symptom index; EDIZ, Ervaren Druk door Informele Zorg [Self-perceived pressure from informal care]; eGFR, estimated glomular filtration rate; GDS-15, geriatric depression scale 15-item; H, Loevinger's coefficient H for scalability; HD, hemodialysis; PD, peritoneal dialysis; HRQoL, health-related quality of life; ICC, intra-class correlation coefficient; LDST, letter-digit substitution test; LOT-R, life orientation test-revised; MCS, mental component score; MoCA, Montreal cognitive assessment; MMSE, mini-mental state examination; NPV, negative predictive value; OR, odds-ratio; PCS, physical component score; PG-SGA, patient-generated subjective global assessment; PPV, positive predictive value; PROMs, patient reported outcome measures; r, correlation coefficient; RR, risk ratio; 6-CIT, 6-item Cognitive Impairment Test; SF-12, 12-item short-form; 95%CI, 95% confidence interval.

**References**

1. Brown EA, Farrington K. Geriatric Assessment in Advanced Kidney Disease. Clin J Am Soc Nephrol. 2019;14:1091-1093

2. Parlevliet JL, Buurman BM, Pannekeet MM, Boeschoten EM, ten Brinke L, Hamaker ME, et al. Systematic comprehensive geriatric assessment in elderly patients on chronic dialysis: a cross-sectional comparative and feasibility study. BMC Nephrol. 2012;13:30

3. Hall RK, Haines C, Gorbatkin SM, Schlanger L, Shaban H, Schell JO, et al. Incorporating Geriatric Assessment into a Nephrology Clinic: Preliminary Data from Two Models of Care. J Am Geriatr Soc. 2016;64:2154-2158

4. Soysal P, Isik AT, Buyukaydin B, Kazancioglu R. A comparison of end-stage renal disease and Alzheimer's disease in the elderly through a comprehensive geriatric assessment. Int Urol Nephrol. 2014;46:1627-1632

5. Berkhout-Byrne N, Kallenberg MH, Gaasbeek A, Rabelink TJ, Hammer S, van Buchem MA, et al. The Cognitive decline in Older Patients with End stage renal disease (COPE) study - rationale and design. Curr Med Res Opin. 2017;33:2057-2064

6. Goto NA, van Loon IN, Morpey MI, Verhaar MC, Willems HC, Emmelot-Vonk MH, et al. Geriatric Assessment in Elderly Patients with End-Stage Kidney Disease. Nephron. 2019;141:41-48

7. Katz S, Ford AB, Moskowitz RW, Jackson BA, Jaffe MW. Studies of illness in the aged. The index of ADL: a standardized measure of biological and psychosocial function. Jama. 1963;185:914-919

8. Hopman-Rock M, van Hirtum H, de Vreede P, Freiberger E. Activities of daily living in older community-dwelling persons: a systematic review of psychometric properties of instruments. Aging Clin Exp Res. 2019;31:917-925

9. Reijneveld SA, Spijker J, Dijkshoorn H. Katz' ADL index assessed functional performance of Turkish, Moroccan, and Dutch elderly. J Clin Epidemiol. 2007;60:382-388

10. Parry SM, Granger CL, Berney S, Jones J, Beach L, El-Ansary D, et al. Assessment of impairment and activity limitations in the critically ill: a systematic review of measurement instruments and their clinimetric properties. Intensive Care Medicine. 2015;41:744-762

11. Gutiérrez-Peredo GB, Martins MTS, da Silva FA, Lopes MB, Lopes GB, Lopes AA. Functional dependence and the mental dimension of quality of life in Hemodialysis patients: the PROHEMO study. Health Qual Life Outcomes. 2020;18:234

12. Hall RK, Rutledge J, Luciano A, Hall K, Pieper CF, Colón-Emeric C. Physical Function Assessment in Older Hemodialysis Patients. Kidney Med. 2020;2:425-431

13. Couderc AL, Boulahssass R, Nouguerede E, Gobin N, Guerin O, Villani P, et al. Functional status in a geriatric oncology setting: A review. J Geriatr Oncol. 2019;10:884-894

14. Puts MT, Santos B, Hardt J, Monette J, Girre V, Atenafu EG, et al. An update on a systematic review of the use of geriatric assessment for older adults in oncology. Ann Oncol. 2014;25:307-315

15. Lawton MP, Brody EM. Assessment of older people: self-maintaining and instrumental activities of daily living. Gerontologist. 1969;9:179-186

16. Jassal SV, Karaboyas A, Comment LA, Bieber BA, Morgenstern H, Sen A, et al. Functional Dependence and Mortality in the International Dialysis Outcomes and Practice Patterns Study (DOPPS). Am J Kidney Dis. 2016;67:283-292

17. Bohannon RW. Test-Retest Reliability of Measurements of Hand-Grip Strength Obtained by Dynamometry from Older Adults: A Systematic Review of Research in the PubMed Database. J Frailty Aging. 2017;6:83-87

18. Rijk JM, Roos PR, Deckx L, van den Akker M, Buntinx F. Prognostic value of handgrip strength in people aged 60 years and older: A systematic review and meta-analysis. Geriatr Gerontol Int. 2016;16:5-20

19. Hwang SH, Lee DH, Min J, Jeon JY. Handgrip Strength as a Predictor of All-Cause Mortality in Patients With Chronic Kidney Disease Undergoing Dialysis: A Meta-Analysis of Prospective Cohort Studies. J Ren Nutr. 2019;29:471-479

20. Tsai YC, Chen HM, Hsiao SM, Chen CS, Lin MY, Chiu YW, et al. Association of physical activity with cardiovascular and renal outcomes and quality of life in chronic kidney disease. PLoS One. 2017;12:e0183642

21. Chang YT, Wu HL, Guo HR, Cheng YY, Tseng CC, Wang MC, et al. Handgrip strength is an independent predictor of renal outcomes in patients with chronic kidney diseases. Nephrol Dial Transplant. 2011;26:3588-3595

22. Park SH. Tools for assessing fall risk in the elderly: a systematic review and meta-analysis. Aging Clin Exp Res. 2018;30:1-16

23. Nasreddine ZS, Phillips NA, Bedirian V, Charbonneau S, Whitehead V, Collin I, et al. The Montreal Cognitive Assessment, MoCA: a brief screening tool for mild cognitive impairment. J Am Geriatr Soc. 2005;53:695-699

24. Tiffin-Richards FE, Costa AS, Holschbach B, Frank RD, Vassiliadou A, Kruger T, et al. The Montreal Cognitive Assessment (MoCA) - a sensitive screening instrument for detecting cognitive impairment in chronic hemodialysis patients. PLoS One. 2014;9:e106700

25. Amatneeks TM, Hamdan AC. Montreal Cognitive Assessment for cognitive assessment in chronic kidney disease: a systematic review. J Bras Nefrol. 2019;41:112-123

26. Ciesielska N, Sokolowski R, Mazur E, Podhorecka M, Polak-Szabela A, Kedziora-Kornatowska K. Is the Montreal Cognitive Assessment (MoCA) test better suited than the Mini-Mental State Examination (MMSE) in mild cognitive impairment (MCI) detection among people aged over 60? Meta-analysis. Psychiatr Pol. 2016;50:1039-1052

27. Jolles J, Houx P, van Boxtel M, Ponds R. The Maastricht Aging Study: Determinants of Cognitive Aging. Maastricht: Neuropsych Publishers; 1995:192.

28. Houx PJ, Shepherd J, Blauw GJ, Murphy MB, Ford I, Bollen EL, et al. Testing cognitive function in elderly populations: the PROSPER study. PROspective Study of Pravastatin in the Elderly at Risk. J Neurol Neurosurg Psychiatry. 2002;73:385-389

29. van Deudekom FJ, Kallenberg MH, Berkhout-Byrne NC, Blauw GJ, Boom H, de Bresser J, et al. Patterns and characteristics of cognitive functioning in older patients approaching end stage kidney disease, the COPE-study. BMC Nephrol. 2020;21:126

30. Katzman R, Brown T, Fuld P, Peck A, Schechter R, Schimmel H. Validation of a short Orientation-Memory-Concentration Test of cognitive impairment. Am J Psychiatry. 1983;140:734-739

31. Tuijl JP, Scholte EM, de Craen AJ, van der Mast RC. Screening for cognitive impairment in older general hospital patients: comparison of the Six-Item Cognitive Impairment Test with the Mini-Mental State Examination. Int J Geriatr Psychiatry. 2012;27:755-762

32. O'Sullivan D, O'Regan NA, Timmons S. Validity and Reliability of the 6-Item Cognitive Impairment Test for Screening Cognitive Impairment: A Review. Dement Geriatr Cogn Disord. 2016;42:42-49

33. O'Sullivan D, Brady N, Manning E, O'Shea E, O'Grady S, N OR, et al. Validation of the 6-Item Cognitive Impairment Test and the 4AT test for combined delirium and dementia screening in older Emergency Department attendees. Age Ageing. 2018;47:61-68

34. Upadhyaya AK, Rajagopal M, Gale TM. The Six Item Cognitive Impairment Test (6-CIT) as a screening test for dementia: comparison with Mini-Mental State Examination (MMSE). Curr Aging Sci. 2010;3:138-142

35. Lucke JA, van der Mast RC, de Gelder J, Heim N, de Groot B, Mooijaart SP, et al. The Six-Item Cognitive Impairment Test Is Associated with Adverse Outcomes in Acutely Hospitalized Older Patients: A Prospective Cohort Study. Dement Geriatr Cogn Dis Extra. 2018;8:259-267

36. Whooley MA, Avins AL, Miranda J, Browner WS. Case-finding instruments for depression. Two questions are as good as many. J Gen Intern Med. 1997;12:439-445

37. Blank K, Gruman C, Robison JT. Case-finding for depression in elderly people: balancing ease of administration with validity in varied treatment settings. J Gerontol A Biol Sci Med Sci. 2004;59:378-384

38. Yesavage JA, Sheikh JI. 9/Geriatric Depression Scale (GDS). Clinical Gerontologist. 1986;5:165-173

39. Dennis M, Kadri A, Coffey J. Depression in older people in the general hospital: a systematic review of screening instruments. Age Ageing. 2012;41:148-154

40. Pocklington C, Gilbody S, Manea L, McMillan D. The diagnostic accuracy of brief versions of the Geriatric Depression Scale: a systematic review and meta-analysis. Int J Geriatr Psychiatry. 2016;31:837-857

41. Scheier MF, Carver CS, Bridges MW. Distinguishing optimism from neuroticism (and trait anxiety, self-mastery, and self-esteem): a reevaluation of the Life Orientation Test. J Pers Soc Psychol. 1994;67:1063-1078

42. Glaesmer H, Rief W, Martin A, Mewes R, Brahler E, Zenger M, et al. Psychometric properties and population-based norms of the Life Orientation Test Revised (LOT-R). Br J Health Psychol. 2012;17:432-445

43. Zawadzka B, Zawadzka S, Bedkowska-Prokop A, Ignacak E, Sulowicz W. Psychological Predictors of Cooperation in the Chronic Treatment of Kidney Transplantation Patients. Transplant Proc. 2016;48:1644-1649

44. Ware J, Jr., Kosinski M, Keller SD. A 12-Item Short-Form Health Survey: construction of scales and preliminary tests of reliability and validity. Med Care. 1996;34:220-233

45. Loosman WL, Hoekstra T, van Dijk S, Terwee CB, Honig A, Siegert CE, et al. Short-Form 12 or Short-Form 36 to measure quality-of-life changes in dialysis patients? Nephrol Dial Transplant. 2015;30:1170-1176

46. Lacson E, Jr., Xu J, Lin SF, Dean SG, Lazarus JM, Hakim RM. A comparison of SF-36 and SF-12 composite scores and subsequent hospitalization and mortality risks in long-term dialysis patients. Clin J Am Soc Nephrol. 2010;5:252-260

47. Pakpour AH, Nourozi S, Molsted S, Harrison AP, Nourozi K, Fridlund B. Validity and reliability of short form-12 questionnaire in Iranian hemodialysis patients. Iran J Kidney Dis. 2011;5:175-181

48. Weisbord SD, Fried LF, Arnold RM, Rotondi AJ, Fine MJ, Levenson DJ, et al. Development of a symptom assessment instrument for chronic hemodialysis patients: the Dialysis Symptom Index. J Pain Symptom Manage. 2004;27:226-240

49. van der Willik EM, Meuleman Y, Prantl K, van Rijn G, Bos WJW, van Ittersum FJ, et al. Patient-reported outcome measures: selection of a valid questionnaire for routine symptom assessment in patients with advanced chronic kidney disease - a four-phase mixed methods study. BMC Nephrol. 2019;20:344

50. Moss AH, Ganjoo J, Sharma S, Gansor J, Senft S, Weaner B, et al. Utility of the "surprise" question to identify dialysis patients with high mortality. Clin J Am Soc Nephrol. 2008;3:1379-1384

51. Downar J, Goldman R, Pinto R, Englesakis M, Adhikari NK. The "surprise question" for predicting death in seriously ill patients: a systematic review and meta-analysis. Cmaj. 2017;189:E484-e493

52. Javier AD, Figueroa R, Siew ED, Salat H, Morse J, Stewart TG, et al. Reliability and Utility of the Surprise Question in CKD Stages 4 to 5. Am J Kidney Dis. 2017;70:93-101

53. Rockwood K, Song X, MacKnight C, Bergman H, Hogan DB, McDowell I, et al. A global clinical measure of fitness and frailty in elderly people. Cmaj. 2005;173:489-495

54. Wallis SJ, Wall J, Biram RW, Romero-Ortuno R. Association of the clinical frailty scale with hospital outcomes. Qjm. 2015;108:943-949

55. Pugh J, Aggett J, Goodland A, Prichard A, Thomas N, Donovan K, et al. Frailty and comorbidity are independent predictors of outcome in patients referred for pre-dialysis education. Clin Kidney J. 2016;9:324-329

56. Alfaadhel TA, Soroka SD, Kiberd BA, Landry D, Moorhouse P, Tennankore KK. Frailty and mortality in dialysis: evaluation of a clinical frailty scale. Clin J Am Soc Nephrol. 2015;10:832-840

57. Charlson ME, Pompei P, Ales KL, MacKenzie CR. A new method of classifying prognostic comorbidity in longitudinal studies: development and validation. J Chronic Dis. 1987;40:373-383

58. Anderson RT, Cleek H, Pajouhi AS, Bellolio MF, Mayukha A, Hart A, et al. Prediction of Risk of Death for Patients Starting Dialysis: A Systematic Review and Meta-Analysis. Clin J Am Soc Nephrol. 2019;14:1213-1227

59. McArthur E, Bota SE, Sood MM, Nesrallah GE, Kim SJ, Garg AX, et al. Comparing Five Comorbidity Indices to Predict Mortality in Chronic Kidney Disease: A Retrospective Cohort Study. Canadian Journal of Kidney Health and Disease. 2018;5:2054358118805418

60. Masnoon N, Shakib S, Kalisch-Ellett L, Caughey GE. What is polypharmacy? A systematic review of definitions. BMC Geriatr. 2017;17:230

61. Ottery FD. Definition of standardized nutritional assessment and interventional pathways in oncology. Nutrition. 1996;12:S15-19

62. Campbell KL, Bauer JD, Ikehiro A, Johnson DW. Role of nutrition impact symptoms in predicting nutritional status and clinical outcome in hemodialysis patients: a potential screening tool. J Ren Nutr. 2013;23:302-307

63. Desbrow B, Bauer J, Blum C, Kandasamy A, McDonald A, Montgomery K. Assessment of nutritional status in hemodialysis patients using patient-generated subjective global assessment. J Ren Nutr. 2005;15:211-216

64. Kosters CM, van den Berg MGA, van Hamersvelt HW. Sensitive and practical screening instrument for malnutrition in patients with chronic kidney disease. Nutrition. 2019;72:110643

65. de Boer AH, Oudijk D, Timmermans JM, Pot AM. [Self perceived burden from informal care: construction of the EDIZ-plus]. Tijdschr Gerontol Geriatr. 2012;43:77-88

66. Pot A, van Dyck R, Deeg D. Perceived stress caused by informal caregiving. Construction of a scale. Tijdschrift voor gerontologie en geriatrie. 1995;26:214-219
